# Reciprocity between New York and New Jersey—A Correction

**Published:** 1910-10

**Authors:** 


### Reciprocity Between New York and New Jersey—a Correction

AN item taken from *The Tribune* published in the August edition of the JOURNAL, relating to the abrogation of the reciprocity treaty between New York and New Jersey, contained some inaccuracies that we desire to correct. The sentence "New York requires three years of high school work as a preliminary to entering a medical school, the fourth year to be made up within the *first two years of the medical course*" is inaccurate in two particulars.

While item 3, Section 166, specifies that "the Regents shall admit to examination any candidate . . . that . . . had *prior* to *beginning second year* of medical study the general education required preliminary to receiving the degree of bachelor or doctor of medicine in this state," item 5 of the same section specifies that "the degree of bachelor or doctor of medicine shall not be conferred in this state before the candidate has filed with the institution conferring it the certificate of the Regents *that before beginning the first annual medical* course counted toward the degree, unless matriculated conditionally as hereinafter specified, he had either . . . completed a full course in a registered academy or a high school . . . A medical school may matriculate conditionally a student deficient in not more than one year's academic work of 15 counts of the preliminary education requirement, provided the name and deficiency of each student so matriculated be filed at the Regents' office within three months after matriculation, and that the deficiency be made up before the student begins the second annual course counted toward the degree."

Under no circumstances are candidates admitted to the licensing examination that have made up the fourth year "within the first two years of the medical course." The New York medical statute contemplated the completion of the preliminary education before the beginning of the professional, and the great majority of students are admitted without condition. The lists of conditional matriculates are required under the provisions of the statute and are carefully checked up by the Department as soon as filed.

It is important that the action of neither of the states be misunderstood, hence, we gladly make this correction.

---

The ravages of Asiatic cholera in Russia, Germany and Italy and the possibility of the plague being brought to this country by infected immigrants have engaged the serious attention of the marine hospital service. Surgeon General Wyman has detailed Surgeon H. R. Carter to visit the plague centers and report by cable. He is now performing that duty.

---

After October 1, 1910, health officers will be required by law to take charge of all persons insane or arrested on charges of insanity. The new law also requires that persons under such arrest must be detained at a place not adjacent to a police station or jail. This will necessitate the abandonment of the new detention room at Station No. 3 in Buffalo. Dr. Fronczak, health commissioner, is preparing new quarters for the detention of the insane who may be arrested by the police.

---

It is announced from Washington that the surgeons of the United States public health and marine hospital service in the Philippines have succeeded in growing the lepra bacillus outside the human body. This achievement, it would appear, is the first step in the production of vaccine or a serum for the cure or prevention of leprosy.

Drs. Brinckerhoff and Curry, and M. T. Hallman, of Honolulu, according to a press dispatch dated August 6. have succeeded in isolating germs of leprosy. This means, it is said, the ultimate discovery of a cure for the disease. The doctors are attempting to make toxin from the bacilli. Experiments at the leper settlement in Honolulu soon will be made.

---

Professor Deycke, of Hamburg, after months of experimental treatment of lepers in British Guiana, announces that a cure discovered by him has been successful. This treatment consists of the subcutaneous injection of nastin—a bacterial fatty body in oily solution, combined with benzoyl chloride. The effect is to destroy the bacilli of leprosy.

At the Bombay Medical Congress last year it was reported by Captain Beauchamp Williams, residency surgeon in the Persian Gulf, that some cases which he had treated by this method appeared to justify Professor Deycke in his claims. The announcement is now made that the Governor of British Guiana has authorised the discharge from the leper asylum of that colony of a negro who had undergone the nastin treatment.

---

Dr. W. A. Evans, Chicago's original and talented Health Commissioner, has issued a new edition of "healthograms," as he calls some of his sanitary rules. "Filth, flies and fever are the three (dis)graces," writes Dr. Evans. He adds these nuggets of advice:

"If it's real estate you're looking for buy 'hokey pokey.' It's 15 per cent. dirt."

"The typhoid chain of F's—filth, fingers, flies, foods, fever."

"Typhoid to prevent costs a few cents, to cure costs many dollars."

"Not every fly that comes along is carrying disease germs, but many of them are, and you can't tell which is which. Take no chances—swat all."

---

"I suppose this crusade against flies is all right, but it's possible to get too much of a good thing," remarked a dining car conductor as he got off his train in Jersey City, as told in the *Tribune*. "You see that odd looking old fellow there? You could spot him for a crank anywhere. He ought to travel in a glass jar. A couple of hours ago he came into my car and I'll bet he cost the company $50. He happened to see one poor lonely little fly buzzing around and he nearly had a fit. I didn't mind that, but he took a little green paper thing out of his pocket—it looked like a torpedo—and lit it. Then, phew, what a smell! It drove everybody else out of the car, but he seemed satisfied. He said it would kill flies. It did, and appetites, too. I chucked it out the window. What d'ye think of an old crank like that, anyway?"

---

THE presentation of anterior poliomyelitis proved an interesting topic at the meeting of the eighth district branch of the Medical Society of the State of New York, Tuesday, September 27, 1910.

It was stated at the afternoon session that twenty-eight cases have been reported to the health department of this city since August 22d. Since then four deaths have occurred, one as recently as the night before the meeting. Dr. Fronczak, health commissioner, told this and urged the coöperation of the physicians to report cases in an effort to check the disease.

Dr. Fronczak said that the spread of the disease led him to require physicians to report all cases. He said that it is often incorrectly diagnosticated for the reason that so few know its real symptoms. He thought that causes for deaths had often been given as Landry's disease, neuritis and partial paralysis when, in reality, it was infantile paralysis.

Dr. L. Kaufmann and Dr. Edward Clark, formerly assistant health commissioner, are a committee previously appointed by the Buffalo Academy of Medicine to collect statistics on the disease. They also participated in the discussion. Dr. Kaufmann's subject was etiology and epidemiology. Dr. Clark sought to show what Massachusetts was doing to keep tabs on the disease, and he wanted New York to keep a better record.

Other papers were: Symptomatology, by Dr. Irving M. Snow; Diagnosis, by Dr. James W. Putnam, and Sequelæ, by Dr. Prescott Le Breton.

---

THE day camp for tuberculosis patients has flourished this year as never before, and the report of the attending physician, Dr. Eckel, shows a large percentage of improvement with the majority of patients, while very few have been discharged because of insubordination. Running a day camp takes patience, system, good management and good judgment to look after the men and women suffering from tuberculosis. There is no opportunity for cutting down the supplies, as a substantial diet is one of the essentials of the treatment, combined with fresh air and freedom from worry.

Unless the present smoke nuisance in North Main street is abated, the camp will have to be moved from its present location at Main street and La Salle avenue. When the wind is in the right direction, the smoke settles down on the camp like a heavy pall, the patients, coughing and with throats irritated and inflamed, suffer materially, and the snowy tents are smudged and blackened. The camp will remain open during October, and the factories responsible for the smoke should abate it without cavil.

---

W. A. EVANS, Chicago, (*Medical Record,* July 30, 1910,) discusses the question of school ventilation. He says the air of school rooms is often too hot and too dry, and the simple addition of 1,800 cubic feet per pupil per hour of fresh air is not effectual ventilation. He holds that the temperature of the air in the school room should not be over 68° F., its humidity should be 60 to 70, and the air should be blown out at stated intervals by raising the windows, the children being at recess. When they return from recess the air is cold, but they have stored heat by exercise, and by the time they have lost this heat the air of the room will be warm again. It is only by a strong draught of air from the open windows that the bacteria in the atmosphere can be blown out.

